# New insights into the roles of lactylation in cancer

**DOI:** 10.3389/fphar.2024.1412672

**Published:** 2024-10-22

**Authors:** Yajun Zhu, Wenhui Liu, Zhiying Luo, Feiyan Xiao, Bao Sun

**Affiliations:** ^1^ Department of Pharmacy, The Second Xiangya Hospital, Central South University, Changsha, China; ^2^ Institute of Clinical Pharmacy, Central South University, Changsha, China; ^3^ Center for Clinical Trial and Research, The Second Xiangya Hospital, Central South University, Changsha, China

**Keywords:** lactylation, lactate, histone and non-histone proteins, cancer, therapeutic targets

## Abstract

Lactylation, a novel discovered posttranslational modification, is a vital component of lactate function and is prevalent in a wide range of cells, interacting with both histone and non-histone proteins. Recent studies have confirmed that lactylation as a new contributor to epigenetic landscape is involved in multiple pathological processes. Accumulating evidence reveals that lactylation exists in different pathophysiological states and leads to inflammation and cancer; however, few mechanisms of lactylation have been elaborated. This review summarizes the biological processes and pathophysiological roles of lactylation in cancer, as well as discusses the relevant mechanisms and potential therapeutic targets, aiming to provide new insights for targeted cancer therapy.

## 1 Introduction

Generally, lactate dehydrogenase (LDH) is common in hypoxic and/or highly glycolytic cells, such as tumors, which converts pyruvate into lactate rather than into the tricarboxylic acid (TCA) cycle (Kes et al., 2020; Ma et al., 2020). The early viewpoint regarded lactate as a metabolic waste, accompanied by diverse harmful effects and related to hypoxia (Ferguson et al., 2018). However, Brooks’s research revealed the process of lactate production in aerobic conditions and how it was used by the body (Brooks, 2009). Additionally, back to the 1920s, Warburg observed that tumors consumed a large amount of glucose, in which about 85% of the ingested glucose was converted into lactic acid, and 5% of pyruvate produced by glycolysis was metabolized by oxidative phosphorylation (OXPHOS) in mitochondria, which promoted the rapid synthesis of ATP in cytoplasm (Vander Heiden et al., 2009). This phenomenon is called aerobic glycolysis, and tumor cells rely on aerobic glycolysis to provide energy even in the presence of sufficient oxygen, which is known as the Warburg effect. With the assistance of monocarboxylic acid transporter (MCT), lactate can be excreted out of the cell together with protons, which can directly result in an increase in the concentration of lactate intracellular and extracellular to eventually form the acidic tumor microenvironment (TME) (Liberti and Locasale, 2016). It is worth mentioning that lactate accumulation is a characteristic of cancer in the tissue microenvironment, and are associated with metastasis and poor clinical outcomes (Certo et al., 2021; Jin et al., 2022). In addition to serving as a fuel substrate, lactate also acts as a precursor that stimulates histone lactylation (Zhang et al., 2019), as well as takes part in crucial biological processes, including macrophage polarization (Irizarry-Caro et al., 2020), glycolysis (Li et al., 2020) and regulation of tumor proliferation (Yu et al., 2021).

Chromatin remodeling and post-translational modification (PTMs), such as ubiquitination, phosphorylation, acetylation, methylation and glycosylation, lead to epigenetic changes (Wang and Wang, 2019), which are usually heritable and reversible, as well as are involved in the occurrence and development of cancer. According to a report in 2019, using lactate as a substrate of lactyl-CoA to promote histone lactylation was a novel PTM, which supported the view that lactylation was directly induced by lactate (Zhang et al., 2019). Similar to other acyl modifications, there are different covalent modification forces, which leads to different degrees of influence of acyl groups on the binding force between histone and DNA (Bowman and Poirier, 2015; Strahl and Allis, 2000; Zhang Y. et al., 2021), finally affecting their function by regulating the expression of genes. Compelling evidence revealed that lactylation played an important role in various types of cancer. Lysine lactylation (Kla) at the K28 site facilitated the proliferation and metastasis of hepatocellular carcinoma (HCC) cells by inhibiting the function of adenylate kinase 2 (AK2) (Yang Z. et al., 2023). Another study showed that increased histone Kla levels might cause upregulation of succinate dehydrogenase and isocitrate dehydrogenase 3 gamma in the TCA cycle, as well as downregulation of hemokinin-1 and pyruvate kinase M in glycolysis, which contributed to attenuating glucose uptake and glycolysis (Jiang et al., 2021). Collectively, lactate and lactylation have attracted increasing attention in the tumorigenesis and TME.

Although lactate and lactylation have received extensive attention, the underlying mechanism remains unclear. This paper discusses the elaborated role of lactate-derived lactylation in cancer and summarizes the relevant mechanisms, aiming to provide new insights for the therapeutic targets of cancer.

## 2 Metabolic heterogeneity in cancer in the context of warburg effects

Generally, the metabolism of cancer cells is considered different from that of normal cells, and the Warburg effect concretizes this difference and is considered to be a hallmark of cancer. Although Warburg effect was a characteristic metabolic feature of cancer cells, recent evidence revealed that the “reverse Warburg effect” (It refers to cancer-associated fibroblasts in tumor stroma that may produce lactate and activate both glycolysis and autophagy) (Pavlides et al., 2010), metabolic symbiosis (Semenza, 2008; Sonveaux et al., 2008) and glutamine metabolism (DeBerardinis and Cheng, 2010) were reported to be the other metabolic features of cancer. A previous study constructed a hybrid multiscale model of tumor growth and highlighted the importance of glycolysis and acidity in the metabolic heterogeneity of cancer (Robertson-Tessi et al., 2015). Moreover, Yu et al. (2017) developed two metabolic signatures, including glycolysis based on the expression of HIF-1 downstream genes and OXPHOS based on the expression of AMPK downstream genes, to predict the different metabolic phenotypes of cancer cells (Yu et al., 2017). With the advancement in the measurement of metabolic feature parameters, it is possible to identify the metabolic heterogeneity in malignant cells. For instance, Xiao et al. (2019) confirmed that variation in OXPHOS activity was the main contributor to the metabolic heterogeneity at single cell resolution in both melanoma and Head and neck squamous cell carcinoma by using single-cell RNA-seq and bulk RNA-seq (Xiao et al., 2019). Recently, a benchmark study utilized machine learning algorithms integrating the omics data into the generic human metabolic model Recon3D and discovered that different pathways, including extracellular transport, peptide metabolism and fatty acid synthesis, etc., might be the main sources for metabolic heterogeneity in cancers (Jalili et al., 2021).

## 3 The biological processes and pathophysiological roles of lactylation

### 3.1 Biological processes of lactylation

**FIGURE 1:**
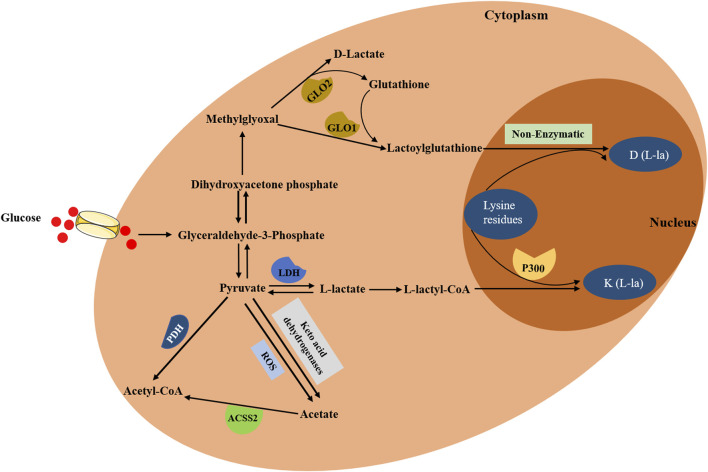
The biological process of lactylation. In the cytoplasm, glucose is transported into cells and is converted to pyruvate and methylglyoxal. On the one hand, pyruvate is converted into L-lactyl-CoA, which then acts as a direct substrate for K (L-la), transferring lactyl groups to lysine residues on histones through histone acetyltransferases such as p300. On the other hand, methylglyoxal is converted to lactoylglutathione, which then generates D (L-la) via nonenzymatic mechanism. Additionally, pyruvate can directly produce acetate through two distinct pathways: keto acid dehydrogenases and ROS-mediated pyruvate decarboxylation. Furthermore, acetate can be converted to acetyl-CoA through ACSS2.

In the cytoplasm, glucose is transported into cells through MCT and is converted to pyruvate and methylglyoxal. Then, the pyruvate is directly reduced to lactate in a process dependent on LDH (Fantin et al., 2006). Lactate typically exists in three forms in mammals, including L-lactate, D-lactate and racemic DL-lactate. The biological process of lactylation may rely on the function of enzymatic and nonenzymatic mechanism (Figure 1). L-lactate, a main form of lactate, can be activated via acyl-CoA synthetase and transported to lysine residues through histone acetyltransferases such as p300, forming a K (L-la) modification on histone proteins (Zhang et al., 2019). Both lactyl-CoA and p300 are needed for enzymatic lysine lactylation; however, there is no evidence to suggest the presence of lactyl-CoA synthase or transferase that converts lactate into lactyl-CoA in mammals. A previous study demonstrated that a propionate CoA-transferase could convert lactate to lactyl-CoA by using a microbial conversion platform in *Escherichia coli* cell (Lee and Trinh, 2019). Furthermore, p300 was also identified to be a lactylation writer protein, and p300 overexpression remarkably increased histone lactylation level (Zhang et al., 2019). Moreover, accumulating studies also identified K (L-la) modification on non-histone proteins (Gao et al., 2020; Hagihara et al., 2021; Meng et al., 2021; Xiong et al., 2022; Yang K. et al., 2022; Zhang N. et al., 2021). In contrast, another study proposed a nonenzymatic mechanism, in which lactyl-glutathione (LGSH) was transferred to lysine residues, generating a K (D-la) modification on non-histone proteins (Gaffney et al., 2020). They also demonstrated that methylglyoxal (MGO), a byproduct of glycolysis, was generated to produce LGSH through glyoxalase 1 (GLO1), and GLO2 hydrolyzed LGSH to produce GSH and D-lactate (Gaffney et al., 2020). Overall, lactate generated in the cytoplasm gives rise to lactyl-CoA and explains the level of histone or non-histone lactylation. That is, lactate acts as a donor for the lactylation of lysine residues, which then directly stimulates or activates the transcription of targeted genes. In addition, histone deacetylases (HDACs), including HDAC1-3 and SIRT1-3, possessed the ability to eliminate Kla (Moreno-Yruela et al., 2022a), among which HDAC3 was the most effective eraser for lactylation (Moreno-Yruela et al., 2022b). Zessin et al. (2022) also proposed that the deacidification activity of HDAC3 was thousands of times higher than that of SIRT2.

Due to the fact that the accumulation of lactate can lead to lactic acidosis (Bennis et al., 2020), lactate requires to be rapidly metabolically removed. Irreversible lactate removal is orchestrated by pyruvate dehydrogenase (PDH), catalyzing the formation of pyruvate and converting the pyruvate to acetyl-CoA (Soreze et al., 2013). In addition, pyruvate can directly produce acetate through two distinct pathways: keto acid dehydrogenases and ROS-mediated pyruvate decarboxylation (Liu et al., 2018). Also, acetate can be converted to acetyl-CoA through acetyl-CoA synthetase 2 (ACSS2), which contributed to TCA cycle, fatty acid synthesis and histone acetylation (Schug et al., 2016). Collectively lactate plays vital roles in regulating metabolic pathways and the process of lactylation.

Although the related effect enzymes of lactic acid have been reported, there is still a gap in the research on the specific mechanism of the process of lactylation. Uncovering these mechanisms will contribute to further understand the significance of its pathophysiological roles.

### 3.2 The main pathophysiological roles of lactylation

#### 3.2.1 Involved in inflammation and macrophage activation and polarization

Inflammation is one of the manifestations of tumor, and persistent inflammation can recruit macrophages. IFNγ activates M1 macrophages, while IL-4 or IL-13 stimulates M2 macrophages (Yunna et al., 2020), which divides macrophages into two functional types. Correspondingly, tumor-associated macrophages (TAM) exert antitumoral and protumoral roles via depending on the activation state, with activated M1 macrophages and alternatively activated M2 macrophages (Mantovani et al., 2002). On the one hand, TAM often shows pro-inflammatory, and anti-tumor activities M1 type in the early stage of carcinogenesis (Wynn et al., 2013). On the other hand, as the tumor growing, M2 phenotype gradually be revealed (Wynn et al., 2013), showing anti-inflammatory, promoting fibrosis, promoting proliferation and angiogenesis activities. High-rate aerobic glycolysis occurs in tumors, during which pro-inflammatory protein synthesis precursors may be produced (Pucino et al., 2017). For instance, studies had found that lactate played an immunosuppressive role in local tissues (Sangsuwan et al., 2020; Xiong et al., 2022). Recently, it had been pointed out that histone lactylation (H3K18la) could promote the expression of protein related to M2 phenotype and accelerate the polarization process of M1 phenotype (Zhang et al., 2019). It was suggested that intracellular lactate levels were increased during the process of the M1 polarization, which was positively correlated with increased histone Kla levels. Further experiment showed that histone lactylation (H3K18la) was enriched at the promoter of arginine enzyme 1 (Arg1), an M2-like gene, and induced Arg1 expression, which confirmed the positive role of H3K18la in driving the expression of protein related to M2 phenotype. Besides, histone lactylation promoted the overexpression of Arg1 and made macrophages turn to anti-inflammatory function (Chu et al., 2021). The alternative view stated that lactylation was a consequence rather than a cause of macrophage activation, but it occurred simultaneously with the metabolic reconnection of IL-6 and Arg1 under inflammatory stress (Dichtl et al., 2021).

In addition to serving as an anti-inflammatory mediator, lactate-derived lactylation may also plays a positive role in macrophage polarization. Treatment of bone marrow-derived macrophages (BMDMs) with lipopolysaccharide (LPS) and IFN-γ can significantly activate Arg1 expression and increase the intracellular levels of lactate and histone Kla (Zhang et al., 2019). After the polarization of M1, BMDMs expressed M2-like phenotype-related genes, such as Arg1 and VEGF. However, BMDMs treated with lactate only showed an elevated lactylation and M2-like phenotype without an early M1 polarization stage. In addition, Dichtl et al. (2021) also proved that there was no obvious correlation between LPS-stimulated Arg1 and histone lactylation. This may indicate that the relationship between Arg1 and histone lactylation is under certain conditions. Further studies are needed to clarify these controversial findings.

Apart from the above pathophysiological roles, lactylation also played a pivotal role in cell fate determination and embryonic development (Li et al., 2020; Tian and Zhou, 2022). Interestingly, the regulatory roles of lactylation in cancer attracted great attention in the field.

## 4 The role of lactylation in cancer

It has been acknowledged that tumor has the advantages of immune escape and metabolic reprogramming, and lactylation induced by lactate (generally acted on histones and non-histones) plays a vital role in various types of cancer by affecting its metabolism and gene expression.

### 4.1 Histone lactylation in different type of cancers

Overwhelming evidence showed that almost each oncogene identified at present had its corresponding target related to regulating metabolism (Levine and Puzio-Kuter, 2010; Locasale et al., 2009; Nagarajan et al., 2016). The abnormality of metabolites also supported the expression of oncogenes and promotes the development of tumors (Li et al., 2018; Liu et al., 2012; Tummala et al., 2017). Histone modification could directly affect the transcription process of genes, leading to the occurrence and development of diseases (Portela and Esteller, 2010). Lactylation is now recognized as the linker to handle metabolism and immune regulation (Chen et al., 2022; Torrini et al., 2022), and the roles of histone lactylation in driving the expression of oncogenes and tumor metabolism deserve more attention.

In ocular melanoma, the level of histone lactylation (H3K18la) was elevated, which contributed to tumorigenesis and poor prognosis by facilitating m^6^A reader protein YTHDF2 expression (Yu et al., 2021). Another study observed that H3K18la drove the activities of key transcription factors YBX1 and YY1 to promote cisplatin resistance in bladder cancer (Li F. et al., 2024). In addition, CircXRN2 was downregulated in bladder cancer through activating Hippo signaling pathway, which further modulated tumor progression by inhibiting H3K18 lactylation (Xie et al., 2023). Inactive von Hippel-Lindau (VHL) was associated with metabolic reprogramming in clear cell renal cell carcinoma (ccRCC), and it was reported that carcinogenic positive feedback exists between histone lactylation caused by inactive VHL in ccRCC and platelet-derived growth factor receptor β (PDGFRβ) (Yang J. et al., 2022). Histone lactylation triggered by inactive VHL could contribute to ccRCC progression through activating the transcription of PDGFRβ, and PDGFRβ can stimulate glycolysis, lactic acid production and the increase of histone lactylation in clear renal cell cancer cells. Nevertheless, the researcher did not find a significant association between histone lactylation and YTHDF2, which suggested that histone lactylation might affect certain genes in a specific system. Jiang et al. found that lactate could down-regulate and up-regulate the expression of glycolytic enzymes (HK-1, PKM) and TCA cycle enzymes (SDHA, IDH3G), respectively, in non-small cell lung cancer (NSCLC), accompanied with the increased histone lactylation (Jiang et al., 2021). They performed further experiments and indicated that lactate regulated this cellular metabolism at least in part via histone lactylation-mediated gene expression. Utilizing bioinformatics analysis, Wang et al. found that the expression of BZW2, a key member of the bZIP superfamily, was increased in lung adenocarcinoma tissues (Wang M. et al., 2023). *In vitro* and *in vivo* studies verified that BZW2 facilitated the malignant progression of lung adenocarcinoma by governing IDH3G expression through glycolysis-mediated histone lactylation. Other studies also found that histone modification sites (H3K9la and H3K56la) were associated with the progress of HCC, and demethylzeylasteral, a triterpene anti-tumor compound, could inhibit H3 histone lactylation and suppress the tumorigenicity (Pan et al., 2022).

**FIGURE 2:**
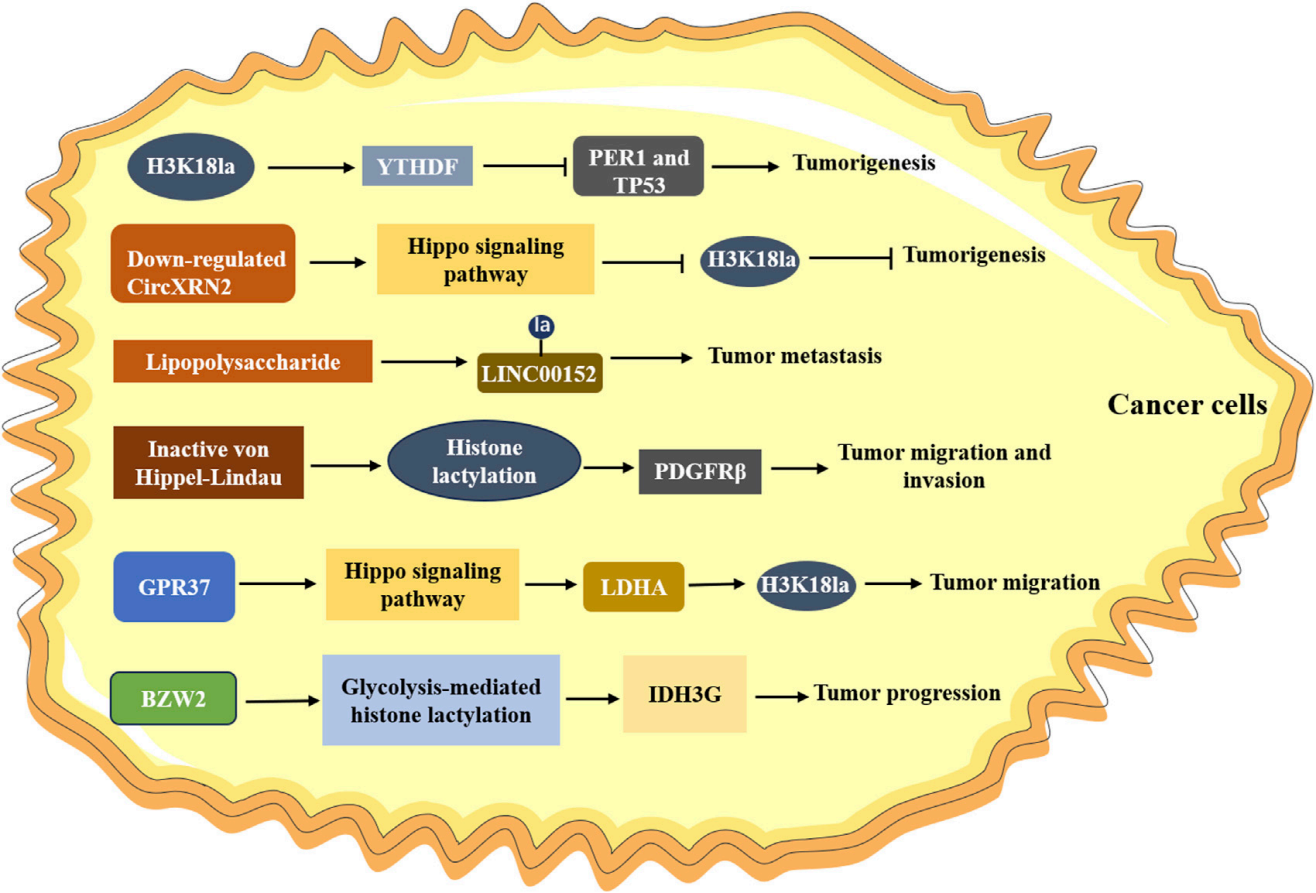
The role of histone lactylation in cancer. H3K18la upregulates the expression of YTHDF, which promoting the degradation of PER1 and TP53, leading to the tumorigenesis; Downregulated CircXRN2 activates the Hippo signaling pathway and promotes tumor progression by inhibiting H3K18 lactylation; Lipopolysaccharide induces lactylation of LINC00152 and promotes the metastasis of tumor; Inactive von Hippel-Lindau promotes cancer progression by activating PDGFRβ through histone lactylation; GPR37 upregulates the expression of LDHA via Hippo signaling pathway, resulting in increasing H3K18la levels in tumors and promoting the metastasis of cancer cells.

Increasing studies reported that histone lactylation played a crucial part in colorectal cancer. Wang et al. (2022) found that LINC00152 was overexpressed in colorectal cancer cells. Further research demonstrated that lipopolysaccharide introduced histone lactylation into the promoter of LINC00152 to promote the metastasis of colorectal cancer. A recent study investigated the global lactylation in colorectal cancer, and identified a 23-gene Lactylation-Related Gene risk model, which was able to predict the prognosis of colorectal cancer patients (Huang et al., 2024). Analogously, Xie et al. (2024) also found that Kla abundance profile was negatively correlated with the prognosis of colorectal cancer, and they identified that KAT8, a lysine acetyltransferase that acted as a pan-Kla writer, could contribute to tumorigenesis through KAT8-eEF1A2 Kla axis. Zhou et al. (2023) supported that the expression of GPR37 was increased in colorectal cancer patients, and mechanism studies revealed that GPR37 promoted LDHA expression and glycolysis via hippo pathway, which resulted in the increased lactylation of H3K18la. Li W. et al. (2024) found higher levels of lactylation and H3K18la in CRC tissues, and H3K18la was correlated with shorter overall survival in CRC patients resistant to bevacizumab treatment. Through cell-based xenografts, patient-derived xenografts and patient-derived organoids models, they revealed that histone lactylation promoted the transcription of RUBCNL/Pacer, accelerating autophagosome maturation through interacting with BECN1/beclin 1. BRAF^V600E^ mutation could trigger a series of signaling cascades essential for proliferation in anaplastic thyroid cancer (ATC) (Shimamura et al., 2020), and it could restructure the cellular lactylation, which contributed to H4K12La-driven gene transcription and thus accelerating ATC proliferation (Wang X. et al., 2023). Recently, a lactylation score model was constructed by using cluster and principal component analysis in gastric cancer, which was involved in increased tumor infiltrating immunocytes and genetic instability, as well as closely associated with the prognosis of gastric cancer (Yang H. et al., 2023). Collectively, these findings underscore the role of histone lactylation in different cancers (Figure 2), and lays the groundwork for mechanism investigation.

### 4.2 Non-histone lactylation in cancers

**FIGURE 3:**
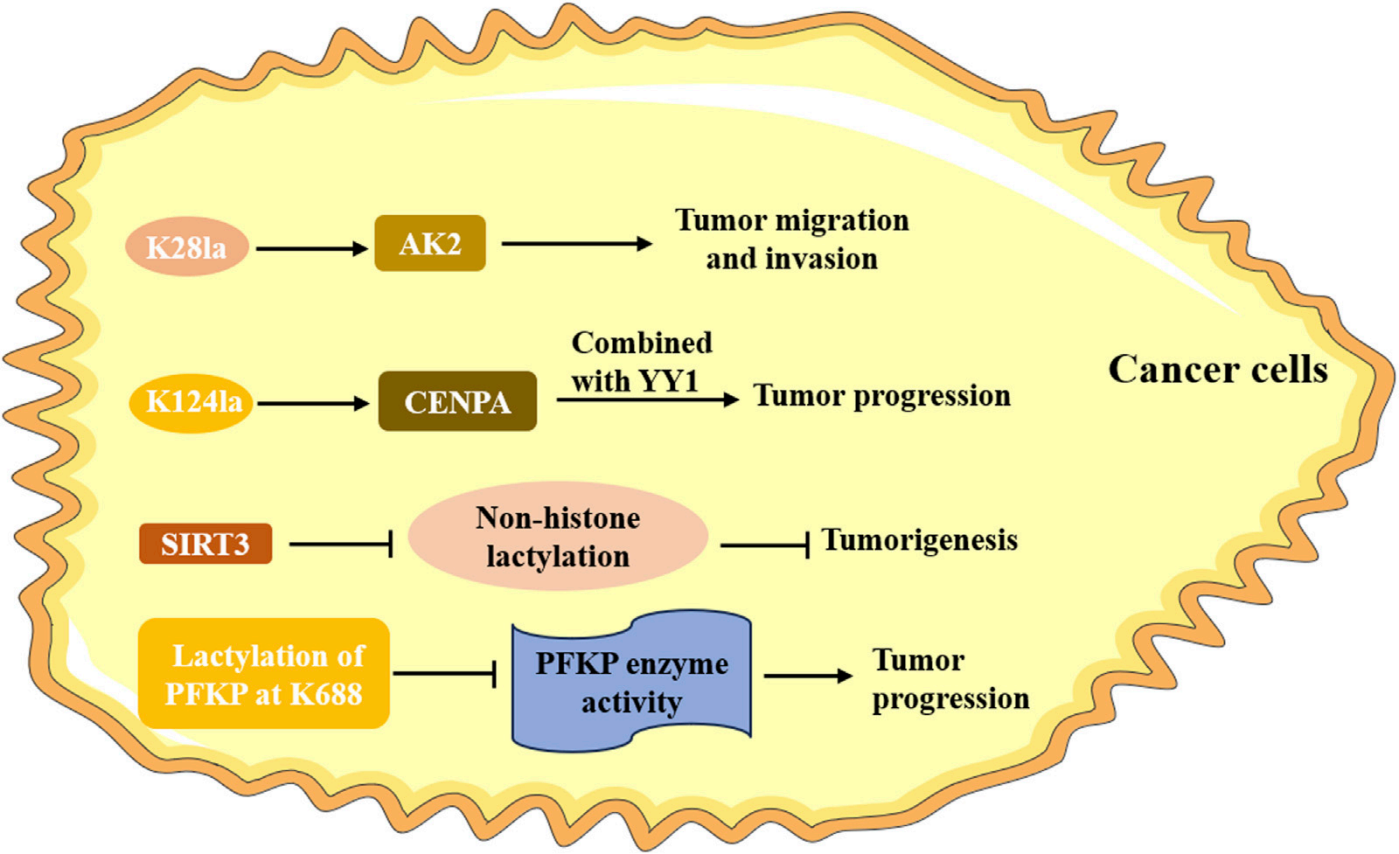
The role of non-histone lactylation in cancer. The lactylation of K28 accelerates the proliferation and metastasis of HCC cells by inhibiting the function of AK2. CENPA is lactylated at the lysine 124, promoting its activation and facilitating tumor progression; SIRT3 can deacetylate non-histone proteins and prevent the occurrence of tumor; the lactylation of the non-histone protein PFKP at K688 directly attenuates it enzyme activity and promotes the progression of tumor.

The specificity and abundance of non-histone proteins are higher than that of histones in cells. Like histone lactylation, non-histone lactylation also played a critical role in cancers (Figure 3). Lactylome profiling analysis not only screened 9,275 histone lactylation sites, but also identified 9,256 non-histone lactylation sites in HCC cells (Yang Z. et al., 2023). It also verified that lactylation at K28 expedited the proliferation and metastasis of HCC cells by inhibiting the function of AK2. SIRT3, mainly as a deacetylase, was negatively correlated with a high level of Kla in HCC tissues and cells, and further experiment suggested that it could deacetylate non-histone proteins and prevent HCC development (Jin et al., 2023). Liao et al. demonstrated that CENPA was highly expressed in HCC, which was correlated with poor prognosis for HCC patients (Liao et al., 2023). Further studies identified that CENPA could be lactylated at lysine 124, which promoted the activation of CENPA, thus enhancing the expression of its target genes. A current study unraveled that copper content promoted non-histone protein METTL16 lactylation at site K229 in gastric cancer through the m^6^A modification, which ultimately led to cuproptosis (Sun et al., 2023). Using mass spectrometry, Cheng et al. (2024) identified 637 lysine lactylation sites in FHC cells and SW480 colon cancer cells. They also investigated that the lactylation of the non-histone protein PFKP at K688 could directly attenuate enzyme activity and thus impacted the glycolysis pathway. Xiong et al. (2022) also found lactylation at non-lysine amino acid sites by using the liquid chromatography-mass spectrum; however, its significant role at these sites is still unclear. Although few studies have focused on non-histone Kla in cancers, the relevant research was insufficient. Further clinical and mechanistic studies are necessary to explore the specific role of non-histone lactylation in cancers.

## 5 Mechanisms of lactylation in tumorigenesis

Mounting research has indicated the critical role of lactylation in cancers, but its mechanism is not fully clear. Here, we discuss the main relevant mechanisms of lactylation in the occurrence and development of tumors (Table 1).

**TABLE 1:** Roles and mechanisms of lactylation in various types of cancer.

Types of cancer	Lactylation sites/proteins	Roles	Mechanisms	References
Ocular melanoma	H3K18la	Accelerated tumorigenesis of ocular melanoma	Facilitated YTHDF2 expression and then promoted the degradation of PER1 and TP53	Yu et al. (2021)
Bladder cancer	H3K18la	Promoted cisplatin resistance in bladder cancer	Activated the transcription of YBX1 and YY1 by enriching in their promoter regions	Li W. et al. (2024)
Bladder cancer	CircXRN2	Inhibited the proliferation and migration of tumor cells	Activated the Hippo signaling pathway	Xie et al. (2023)
ccRCC	Global histone lactylation	Promoted the progression of clear cell renal cell carcinoma	Activated the transcription of PDGFRβ	Yang K. et al. (2022)
NSCLC	HK-1 and IDH3G	Associated with the poor prognosis of non-small cell lung cancer	Downregulated HK-1 expression and upregulated IDH3G expression by enriching histone lactylation in their promoter regions	Jiang et al. (2021)
Lung adenocarcinoma	IDH3G	Facilitated the malignant progression of lung adenocarcinoma	Promoted glycolysis-mediated lactate production and lactylation of IDH3G	Wang J. H. et al. (2023)
HCC	Lactylation of K28	Promoted the proliferation and metastasis of HCC cells	Inhibited the function of AK2 and affected the biological behavior of cancer cells	Yang M. et al. (2023)
Colorectal cancer	LINC00152	Promoted the migration and invasion of colorectal cancer cells	Introduced histone lactylation into the promoter of LINC00152 and increased the expression of LINC00152	Wang et al. (2022)
Colorectal cancer	KAT8	Contributed to tumorigenesis	Resulted in boosted translation elongation and enhanced protein synthesis	Xie et al. (2024)
Colorectal cancer	H3K18la	Correlated with shorter overall survival	Promoted the transcription of RUBCNL/Pacer and accelerated autophagosome maturation	Li F. et al. (2024)
ATC	BRAF^V600E^	Accelerated the proliferation of ATC	Activated the expression of multiple genes essential for ATC proliferation	Shimamura et al. (2020)
HCC	SIRT3	Suppressed the development of HCC	Deacetylated non-histone proteins and prevented HCC development	Jin et al. (2023)
HCC	CENPA lactylation at site K124	Correlated with poor prognosis for HCC	Functioned as a transcriptional regulator to promote HCC via cooperating with YY1	Liao et al. (2023)
Gastric cancer	METTL16 lactylation at site K229	Acted as a driving force for cuproptosis	Accelerated dihydrolipoamide S-acetyltransferase lipoylation and cuproptosis	Sun et al. (2023)
Colorectal cancer	PFKP at site K688	Associated with the development of colorectal cancer	Directly attenuated enzyme activity and impact the glycolysis pathway	Cheng et al. (2024)
PDAC	NUSAP1	An independent unfavorable predictor of PDAC prognosis	Bound to c-Myc and HIF-1α and enhanced its expression through Kla modification	Chen et al. (2023)
Prostate cancer	KIAA1199	Promoted angiogenesis and vasculogenic mimicry	Stabilized HIF-1α that acted as the transcription enhancer of KIAA1199 via HIF-1α lactylation	Luo et al. (2022)
Lung cancer and gastric cancer	ULK1	Maintained muscle cell homeostasis and correlated with cancer progress	Directly interacted with LDHA and promoted autophagic flux and endolysosomal trafficking through Vps34 lactylation	Jia et al. (2023)

ccRCC, clear cell renal cell carcinoma; NSCLC, non-small cell lung cancer; HCC, hepatocellular carcinoma; ATC, anaplastic thyroid cancer; PDAC, pancreatic ductal adenocarcinoma; LDHA, lactate dehydrogenase A.

### 5.1 Involved in the metabolism of tumor cells

It is noteworthy that metabolic reprogramming of tumor cells promotes the progress of tumor through regulating the metabolic enzymes, channels, and other associated genes (Gao et al., 2019; Jiang et al., 2019; Martínez-Reyes and Chandel, 2021). Lactylation is now considered to be involved in the metabolism of tumor cells by driving the expression of metabolic enzymes and genes. For instance, Kla at the K147 site of fructose-bisphosphate ALDOA caused enzyme inhibition, which influenced the process of glycolysis (Wan et al., 2022). Nucleolar and spindle associated protein 1 (NUSAP1), a microtubule-associated protein, played an important role in metastasis of pancreatic ductal adenocarcinoma (PDAC) by regulating LDHA-mediated glycolysis (Chen et al., 2023). Mechanically, NUSAP1 bound to c-Myc and HIF-1α, which then formed a transcription regulatory complex (localized to LDHA promoter region) and enhanced the expression of LDHA. Kla was also reported to affect enzymes involved in metabolic pathways such as the TCA cycle, amino acid, glucose and nucleotide metabolism, which then inhibited AK2 and promoted the growth of liver cancer cells (Yang Z. et al., 2023). Another study demonstrated that increased histone Kla downregulated HK-1 and PKM in glycolysis, and upregulated SDHA and IDH3G in the TCA cycle (Jiang et al., 2021). Until now, there are few investigations of metabolic pathways. Thus, further exploration of the mechanism of lactylation in tumor metabolism are needed to provide more potential targets for tumor therapy.

### 5.2 Modulated the TME

**FIGURE 4:**
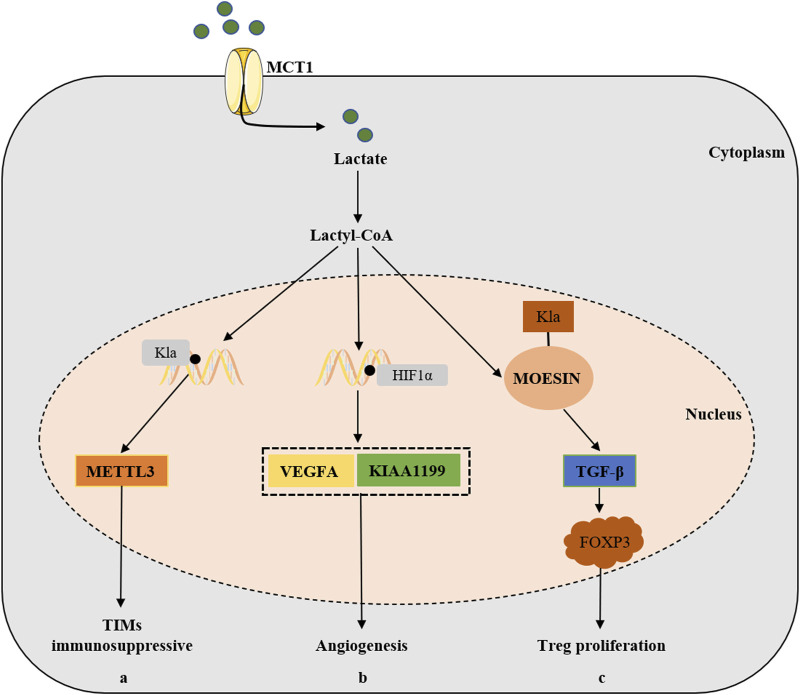
The relevant mechanisms of lactylation in the development of cancer by modulating TME. Lactate can also be transported into cells via MCT1 and is converted to lactoyl-CoA, (a) which stimulates the expression of METTL3 in TIMs via H3K18 Kla lactylation and ultimately promotes the immunosuppressive capacity of TIMs. (b) Lactate accumulation and Kla promotes the expression of VEGFA and KIAA1199 via HIF-1α lactylation, thus participating in tumorigenesis via angiogenesis pathways. (c) Lactylation of MOESIN protein upregulates TGF-β signaling in Treg cells, thereby promoting the proliferation of Treg cells and enhancing the tumorigenesis.

TME includes a variety of cell types, such as immune cells, cancer-associated fibroblasts (CAFs), endothelial cells, growth factors, extracellular matrix (ECM), and cellular metabolites related to hypoxia and acidity, which is the critical mediator in carcinogenesis. As one of the most significant metabolites in TME, lactate produced by tumor cells through the Warburg effect, which could promote angiogenesis and regulate the metabolism of immune cells, as well as mediate immune escape (Brown and Ganapathy, 2020; Tu et al., 2021), providing an important underlying condition for lactylation (Bader et al., 2020; Wang J. H. et al., 2023). On the other hand, the level of histone lactylation has been found to be higher in TME, implying that it plays an important regulatory role in pathogenesis of tumor (Figure 4).

Vascular endothelial growth factor A (VEGFA) is the main driver of angiogenesis, and HIF-1α is the transcription enhancer of VEGFA (Ferrara and Kerbel, 2005). Lactate accumulation and Kla were involved in angiogenesis through VEGFA and HIF-1α (Morland et al., 2017; Young et al., 2014). KIAA1199, a hyaluronic acid (HA)-binding protein, was positively correlated with the angiogenesis markers in cholangiocarcinoma (Zhai et al., 2020), and was also found to promote angiogenesis and vasculogenic mimicry in prostate cancer (Luo et al., 2022). Further experiments revealed that lactate stabilized HIF-1α that acted as the transcription enhancer of KIAA1199 via HIF-1α lactylation, which suggested that lactylation participated in tumorigenesis via angiogenesis pathways.

The lactylation was not involved in a single cell type of TME, and immune cells also played an important role. As already mentioned earlier, prior studies indicated that histone lactylation were positively correlated with the oncogenicity of M2 macrophages (Zhang et al., 2019). Tumor infiltrating myeloid cells (TIMs) were also an important cell type involved in tumor immune escape, and their function was regulated through multiple epigenetic mechanisms. Wang et al. found that lactate stimulated the expression of METTL3 in TIMs via H3K18 Kla lactylation, which was associated with poor prognosis in colon cancer patients (Lan et al., 2021). They further found that the accumulated lactate in TME could promote the upregulation of METTL3 via H3K18 lactylation in TIMs. Furthermore, two lactylation modification sites were identified in the zinc finger structural domain of METTL3, which was critical for METTL3 to capture target RNAs, thus emphasizing the significance of lactylation-driven METTL3-mediated RNA m^6^A modifications for promoting the immunosuppressive capacity of TIMs (Xiong et al., 2022). It’s well known that regulatory T (Treg) cells play a significant role in reducing the activity of anti-tumor T cells and maintaining the immunosuppressive microenvironment (Kasagi et al., 2014). Studies had shown that lactate could promote the generation and function of Treg cells through the lactylation of Lys72 in MOESIN, and then enhance TGF-β signaling to up-regulate the expression of FOXP3, thus promoting the tumorigenesis (Gu et al., 2022).

Apart from the above potential mechanisms, autophagy was also involved in the pathogenesis of cancer through lactylation. Autophagy had been proved to remove damaged organelles and protein to promote tumor development, improve the metabolic adaptability of malignant cells and improve the survival rate (Kimmelman and White, 2017; Miller and Thorburn, 2021; Yang et al., 2018). Jia et al. (2023) revealed that lactate connected autophagy and glycolysis through Vps34 lactylation, which further promoted autophagic flux and endolysosomal trafficking, ultimately leading to cancer progress. In recent years, gut microbiome has been recognized as an important factor in regulating the development of cancer via targeting lactate and lactylation pathways. For instance, bacteria-derived lipopolysaccharide introduced histone lactylation on the promoter of LINC00152, thereby upregulating its expression and promoting tumor growth of CRC (Wang et al., 2022). Therefore, further exploration of the specific mechanisms of lactylation may provide more potential therapeutic targets for cancer treatment.

## 6 The potential therapeutic targets of lactylation in cancer

Previous studies have identified lactate as a novel oncotherapeutic target (Spencer and Stanton, 2019; Taddei et al., 2020). Similarly, lactylation played a vital role in cancer and was also expected to be used as a therapeutic target. Indeed, targeting lactylation could inhibit cancer progression and enhance antitumor effects (Fan et al., 2023). Particularly, drugs targeting the process of lactylation have achieved remarkable success in clinical applications of antitumor therapy. For instance, Oxamate, a competitive inhibitor of LDH, inhibited lactate production and lactylation modifications, thus affecting cellular glycolysis (Xing et al., 2018). Several studies reported that oxamate could hinder the growth of cells in lymphoma and nasopharyngeal and gastric cancer (Lin et al., 2022; Yu et al., 2020). In addition, FX11, a selective inhibitor of LDHA, was observed to inhibit the proliferation of cancer cells (Lin et al., 2022) and exert antitumor activity in a mouse transplantation tumor model (Mohammad et al., 2019). In recent years, some specific compounds, including phthalimide, dibenzofuran derivatives and 1-(phenylseleno)-4-(trifluoromethyl) benzene, were shown to function as LDHA inhibitors and displayed the inhibition of the development and multiplication of cancer cells (Kim et al., 2019). Intriguingly, a previous study found that siRNAs specifically targeting LDHA demonstrated the inhibition of cancer progress, thereby showing a potential therapeutic strategy for LDHA-dependent malignancies (Daneshmandi et al., 2019). Collectively, these discoveries provide a solid basis for LDHA inhibitors targeting the process of lactylation in the treatment of cancer.

Targeting the lactate transporter protein MCTs has also been reported to inhibit the migration, invasion and metastasis of cancer cells (Sprowl-Tanio et al., 2016), which provides a promising strategy for cancer treatment. Numerous MCT inhibitors, such as α-cyano4-hydroxycinnamatem, lonidamine, simvastatin, quercetin and phloretin have exhibited therapeutic potential for various cancer types, including breast cancer, colorectal cancer, cervical cancer and prostate cancer, etc. (Ghafouri-Fard et al., 2021; Kirkland and Tchkonia, 2020). Notably, there were also studies on MCT1-targeted drug AZD3956 that was currently in clinical trials (NCT01791595). Although increasing evidence identified lactylation as a therapeutic target to impede cancer progress and restore tumor sensitivity to treatment, it is essential to explore the novel mechanism for responding to the critical role of lactylation in tumorigenesis and provide new targets for tumor therapy.

## 7 Conclusion and future perspective

We have attempted to summarize the biological processes of lactylation and discussed its critical pathophysiological roles in the occurrence and progression of cancer. We also generalized the potential therapeutic targets of lactylation in the treatment of cancer. These findings highlight the important role of lactylation in tumor patients and provide the essential help for clinicians to manage them.

Although accumulating evidence underscored the crucial role of lactylation in cancer, studies on lactylation are still in their infancy. Importantly, the association between lactylation and tumorigenesis required systematically analyzed. With the development of high-throughput sequencing technology, the features from extensive cancer data were investigated, which facilitated pan-cancer analysis. A recent study provided a comprehensive overview of lactylation genes at the pan-cancer level and assessed the correlation of lactylation scores with clinical features, TME and prognosis by integrating multiple computational methods (Wu et al., 2024). At present, few lactylation sites had been found, and search for more these sites could provide more reliable targets for tumor therapy. Sun et al. (2022) developed a bioorthogonal chemical reporter, YnLac, which could enable the identification of lactylation sites in combination with chemical proteomics in mammalian cells, opening a new avenue for identifying PTM. Auto-Kla, a novel computational model, was proposed by Lai and Gao (2023), which could quickly and accurately predict Kla sites based on automated machine learning and provide a useful analytical tool for PTM prediction. In short, the integration of machine learning algorithms with multi-omics approaches could provide multi-faceted insight into the role of lactate and lactylation in cancer and deepen our understanding of the underlying mechanisms. On the other hand, as mentioned earlier, Lu et al. indicated that Kla levels in the Treg cells of HCC patients who responded to anti-PD-1 therapy were lower, and cotreatment with anti-PD-1 drugs and lactate dehydrogenase inhibitors has a stronger antitumor effect than anti-PD-1 drugs alone (Gu et al., 2022). This result address the necessary to investigate the effect of Kla on immune cells for improving the safety and effectiveness of immunotherapy in the future.

Taken together, unraveling the role and mechanisms of lactylation in a range of cancers will be conducive to taking targeted measures in clinical practice.

## Glossary

LDH: lactate dehydrogenase;
TCA: tricarboxylic acid
OXPHOS: oxidative phosphorylation
MCT: monocarboxylic acid transporter
TME: tumor microenvironment
PTMs: post-translational modification
Kla: Lysine lactylation
HCC: hepatocellular carcinoma
AK2: adenylate kinase 2
LGSH: lactyl-glutathione
MGO: methylglyoxal
GLO1: glyoxalase 1
HDACs: histone deacetylases
PDH: pyruvate dehydrogenase
ACSS2: acetyl-CoA synthetase 2
TAM: tumor-associated macrophages
Arg1: arginine enzyme 1
BMDMs: bone marrow-derived macrophages
LPS: lipopolysaccharide
AD: Alzheimer’s disease
VHL: von Hippel-Lindau
ccRCC: clear cell renal cell carcinoma
PDGFRβ: platelet-derived growth factor receptor β
NSCLC: non-small cell lung cancer
ATC: anaplastic thyroid cancer
PDAC: pancreatic ductal adenocarcinoma
CAFs: cancer-associated fibroblasts
ECM: extracellular matrix
VEGFA: vascular endothelial growth factor A
HA: hyaluronic acid
TIMs: Tumor infiltrating myeloid cells;
Treg: regulatory T.
